# Fc Gamma Receptor IIb Expressed in Hepatocytes Promotes Lipid Accumulation and Gluconeogenesis

**DOI:** 10.3390/ijms19102932

**Published:** 2018-09-26

**Authors:** Ting Shu, Xiaomin Song, Xingxing Cui, Weipeng Ge, Ran Gao, Jing Wang

**Affiliations:** State Key Laboratory of Medical Molecular Biology, Institute of Basic Medical Sciences, Chinese Academy of Medical Sciences, Department of Pathophysiology, Peking Union Medical College, Beijing 100005, China; 15901356250@126.com (T.S.); 18271392658@163.com (X.S.); cuixingxing90@163.com (X.C.); 18840827758@163.com (W.G.); gaoran@ibms.pumc.edu.cn (R.G.)

**Keywords:** FcγRIIb, NAFLD, gluconeogenesis, insulin sensitivity

## Abstract

Non-alcoholic fatty liver disease (NAFLD) is characterized by ectopic lipid accumulation in the liver, usually combined with hepatic insulin resistance. Fc-gamma receptor-IIb (FcγRIIb) and its ligand are reported to be associated with obesity and type 2 diabetes mellitus (T2DM). As knowledge about FcγRIIb in the literature is mostly generated from studies on skeletal muscle tissue, the expression and function of FcγRIIb in the liver and hepatocytes are largely unknown. In this study, we identified the expression of FcγRIIb in primary cultured mouse hepatocytes: FcγRIIb was upregulated in response to oleic acid (OA) in a dose dependent manner. FcγRIIb knockdown using shRNA suppressed the lipid and triglyceride accumulation, and mRNA expression of *ACC1*, *FASn*, *CD36*, *MTTP*, and *ApoB* in OA-treated HepG2 cells. FcγRIIb deficiency mice fed with high fat diet (HFD) had significantly lower liver weight and liver to body weight ratio, as well as less triglyceride accumulation in the livers. In glycometabolism, FcγRIIb hindered insulin-induced phosphorylation of AKT and FOXO1, and in turn upregulated *G6Pase* and *PEPCK* mRNA expression, suggesting that FcγRIIb promotes gluconeogenesis by suppressing the AKT/FOXO1/*G6Pase*/*PEPCK* pathway in hepatocytes. This study reveals a novel role for FcγRIIb in regulating lipid metabolism and glycometabolism, and provides a new therapeutic target to improve NAFLD.

## 1. Introduction

Non-alcoholic fatty liver disease (NAFLD) is a progressive liver disease. Patients with NAFLD have ectopic lipid accumulation in liver, and are in a high risk of developing into non-alcoholic steatohepatitis (NASH), cirrhosis, hepatocellular carcinoma (HCC), and hepatic insulin resistance [1]. The prevalence of NAFLD reached 25–30% worldwide, and 30–40% among them will get worse [2]. The main cause of NAFLD is the over nutrition and obesity. As a nexus of metabolic and hepatic disease [3], liver damage will cause hyperglycemia, hyperlipidemia, and type 2 diabetes mellitus (T2DM).

The liver is an organ in charge of the balance of energy storage and supply. Food intake evokes high concentration of insulin that activates insulin receptor. Activated insulin receptor promotes phosphorylation of AKT and its downstream factor, FOXO1 in hepatocyte [4]. As a transcription factor, phosphorylation of FOXO1 suppresses its translocation into nuclear where it is supposed to upregulate expression of *G6Pase* and *PEPCK* [5]. G6Pase and PEPCK are important enzymes for gluconeogenesis, which restrain hyperglycemia [3,4]. Liver also makes blood glucose stable in fasting state. However, under insulin resistance, accumulation of free fatty acid (FFA) in the liver, and high concentration of glucose in blood will occur [6]. Liver de novo lipogenesis and fatty acid esterification are the two main sources that promote lipid synthesis in hepatocyte [2,3,7]. Under normal physiological condition, glucose from blood is taken up into the hepatocyte to be synthesized into triglyceride by de novo lipogenesis related factors *SREBP-1*, *ACC1* and *FASn* [3]. Serum FFA is transported into the hepatocyte by fatty acid transport proteins (such as *CD36*) and fatty acid binding proteins in the membrane. Intracellular fatty acid is esterified it into triglyceride [2]. Lipolysis also controls lipid accumulation in hepatocytes through fatty acid β-oxidation in mitochondria [8]. Nuclear receptor *PPARα* controls every enzymatic step related gene within the fatty acid oxidation pathways, such as *CPT1α* and *MCAD* [8]. Suppression of *PPARα* makes fatty acid fail to import into the mitochondria, which results in low lipolysis and lipid accumulation in the hepatocyte [8].

FcγRIIb, an Immuneglobulin G(IgG) receptor, is a tyrosine kinase receptor, located at the cell membrane. FcγRIIb is the only inhibitory receptor of IgG [9]. FcγRIIb transmits inhibitory signals through an immunoreceptor tyrosine-based inhibitory motif (ITIM) in its cytoplasmic region. Activation of FcγRIIb triggers ITIM phosphorylation, and results in recruitment of phosphatases including a SH2 domain containing inositol polyphosphate 5′ phosphatase (SHIP) and a SH2-domain containing protein tyrosine phosphatase 1 [10]. Previous studies demonstrated that FcγRIIb was associated with T2DM and obesity. Tanigaki reported that FcγRIIb was expressed in skeletal muscle microvascular endothelium [11], and regulated endothelium insulin delivery and skeletal muscle glucose uptake using C Reactive Protein (CRP) transgenic mice and endothelial cell-specific FcγRIIb knockout mice [12]. Tanigaki also reported that IgG from patients with T2DM and High Fat Diet-fed mice exhibited hyposialylation in its Fc glycan, which resulted in blunting endothelial insulin transcytosis [13]. However, it is unknown whether the FcγRIIb ligand and its receptor axis existed in hepatocytes and if so, how it regulated hepatocyte function.

In our current study, we identified the expression of FcγRIIb in hepatocytes, and the novel role for FcγRIIb in regulating hepatocytic lipid and glucose metabolism in vivo and in vitro. Our data suggest that FcγRIIb promotes lipid accumulation and glyconeogenesis, which favor development of NAFLD and insulin resistance.

## 2. Results

### 2.1. FcγRIIb Expressed in Hepatocytes

**Figure 1:**
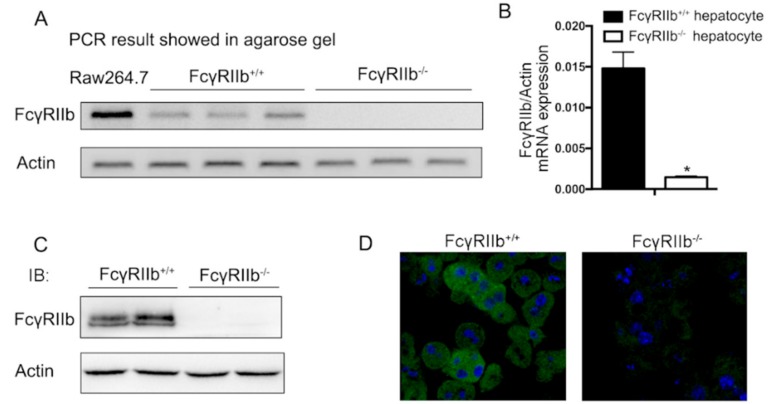
Expression of FcγRIIb in hepatocytes. Primary cultured mouse hepatocytes were isolated from FcγRIIb^+/+^ and FcγRIIb^−/−^ mice. (**A**) RNA was extracted and RT-PCR was performed using primers designed for amplification of FcγRIIb. Macrophage celline RAW264.7 was used as a positive control. (**B**) *FcγRIIb/actin* mRNA expression ratio, detected by qRT-PCR. * *p* < 0.05, *n* = 3 (**C**) Western blot for FcγRIIb protein expression in primary cultured mouse hepatocyte. (**D**) ICC staining of FcγRIIb in primary cultured mouse hepatocyte (40×). Positive area is shown in green, DAPI is shown in blue.

To assess if the FcγRIIb ligand and its receptor axis play a role in hepatocytes, we analyzed *FcγRIIb* expression in isolated hepatocytes. Quantitative RT-PCR analysis showed that *FcγRIIb* mRNA was expressed in hepatocytes compared with the FcγRIIb positive cell, RAW264.7, and that the mRNA level of *FcγRIIb* was markedly higher in primary cultured hepatocytes from wildtype(FcγRIIb^+/+^) mice than that in hepatocyte from FcγRIIb knockout(FcγRIIb^−/−^) mice (Figure 1A,B). Consistent with our qRT-PCR result, the level of FcγRIIb protein was also markedly elevated in hepatocytes from FcγRIIb^+/+^ mice detected in Western blot (Figure 1C). To examine the existence of FcγRIIb in intact hepatocytes, we performed immunofluorescence cell staining for FcγRIIb in primary cultured hepatocytes. The results showed intensively positive staining with FcγRIIb^+/+^ hepatocytes but not with FcγRIIb^−/−^ hepatocytes (Figure 1D). These data suggest that FcγRIIb is expressed in hepatocytes.

### 2.2. Increase of FcγRIIb Expression after Oleic Acid Treatment

**Figure 2:**
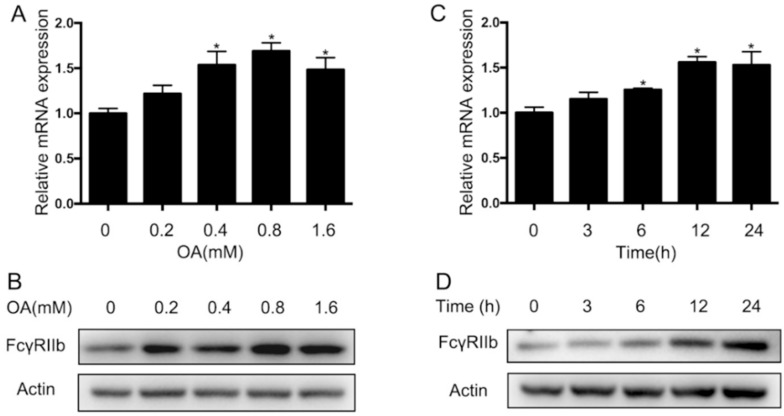
FcγRIIb expression and regulation in HepG2. (**A**) qRT-PCR for mRNA expression of *FcγRIIb* in HepG2 in response to the indicated doses of oleic acid (OA) for 24 h. (**B**) Western blot analysis for FcγRIIb expression under the same conditions as described in A. (**C**) qRT-PCR for mRNA expression of *FcγRIIb* in HepG2 cells in response to 0.4 mM OA treatment for indicated periods. (**D**) Western blot analysis for detecting FcγRIIb expression under the indicated conditions. The data shown are the means ± SEM. * *p* < 0.05.

To explore the role of FcγRIIb in regulating hepatocyte lipid metabolism, we examined the expression of FcγRIIb expression in response to oleic acid (OA) in human hepatocarcinoma cell line HepG2, as OA was used to induce triglyceride accumulation [14]. After 24 h incubation with OA, the expression of *FcγRIIb* in HepG2 was detected by qRT-PCR and Western blot. The results showed that *FcγRIIb* expression in HepG2 was significantly upregulated in HepG2 treated with 0.4 mM OA, and peaked with 0.8 mM OA treatment at both mRNA and protein levels (Figure 2A,B). When treated with 0.4 mM OA, *FcγRIIb* expression in HepG2 plateaued at 12 h after the OA treatment (Figure 2C,D). These results indicated that *FcγRIIb* was upregulated in response to OA treatment, and suggest that *FcγRIIb* may play a role in hepatocyte lipid metabolism.

### 2.3. Increase of FcγRIIb Expression after Oleic Acid Treatment

**Figure 3:**
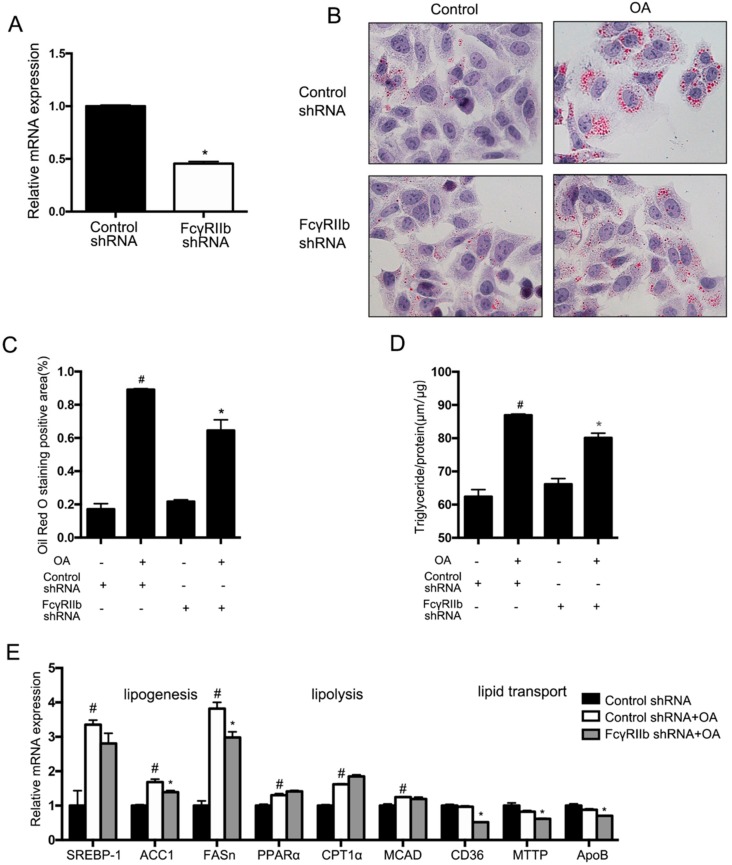
Effect of *FcγRIIb* knockdown on lipid accumulation in OA induced steatosis. (**A**) Relative mRNA expression of *FcγRIIb* in HepG2 48 h after FcγRIIb-shRNA and Control-shRNA administration. (**B**) Oil Red O staining (40×) for lipid droplets in HepG2 after 24 h OA treatment. (**C**) Statistical analysis of Oil Red O staining positive areas. * *p* < 0.05, between control-shRNA and FcγRIIb-shRNA treatment under OA, ^#^ *p* < 0.05, between basal and OA treament in control-shRNA. (**D**) Measurement of intracellular triglyceride content in HepG2. * *p* < 0.05, between control-shRNA and FcγRIIb-shRNA treatment under OA, ^#^ *p* < 0.05, between basal and OA treatment in control-shRNA transfected. (**E**) Relative mRNA expression of the genes involved in lipid synthesis, β-oxidation, and lipid transportation in control cell of basal level, knockdown, and control HepG2 after OA treatment. Gene expression was normalized to that of β-actin. The data shown are the means ± SEM. ^#^ *p* < 0.05, between control shRNA cell with and without OA treatment (bar 1 and bar 2); * *p* < 0.05, between control and FcγRIIb shRNA with OA treatment (bar 2 and bar 3).

To verify the role of FcγRIIb in hepatocyte lipid accumulation, HepG2 cells were transfected with lentivirus expressing FcγRIIb shRNA or a control shRNA. With 48 h transfection, *FcγRIIb* mRNA in the cells treated with FcγRIIb shRNA-lentivirus was significantly decreased compared to the control shown in qRT-PCR (Figure 3A). Afterward, HepG2 cells transfected as described above were incubated with or without OA for 24 h. Oil Red O staining showed no difference in lipid accumulation between the cells transfected with FcγRIIb shRNA and control shRNA in basal level, but significantly fewer lipid droplets in the cells with FcγRIIb knockdown compared with the control after OA treatment (Figure 3B,C). In accord with the Oil Red O staining, the total triglyceride assay showed significantly less triglyceride content in the cells with FcγRIIb knockdown (Figure 3D). These results indicate that FcγRIIb plays a role in mediating OA-induced lipid accumulation in hepatocyte.

To elucidate the targets of FcγRIIb, qRT-PCR was performed to evaluate the putative genes involved in lipogenesis, fatty acid oxidation, and lipid transportation (Figure 3E) in control HepG2 cells with or without OA treatment, and knockdown FcγRIIb with OA treatment. The results showed that OA-treated HepG2 cells exhibited a marked increase in mRNA expression of the indicated lipogenesis genes (Figure 3E, the three groups in the left) and a mild increase in that of the lipolysis genes of interest (Figure 3E, the three groups in the middle), which favors lipid metabolism in the cells. However, knockdown of FcγRIIb significantly resulted in reversions in the OA-induced upregulation of lipogenesis mRNAs compared to their controls (*ACC1* and *FASn*), and lipid transport gene (*CD36*, *MTTP* and *ApoB*), but showed no inhibiting effect on expression of the indicated lipolysis genes (*PPARα*, *CPT1α* and *MCAD*). These results suggest that FcγRIIb promotes lipid accumulation in hepatocytes through enhancing lipogenesis and lipid transportation.

### 2.4. FcγRIIb-Deficiency Attenuated HFD-Induced Hepatic Steatosis in Mice

**Figure 4:**
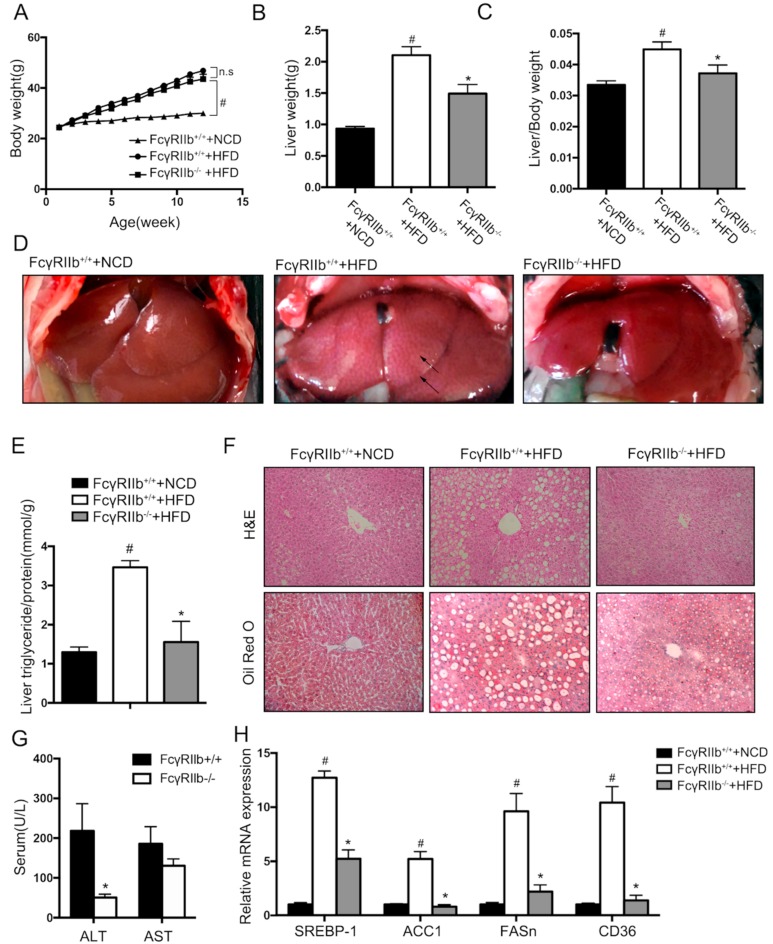
Effect of FcγRIIb knockdown on high fat diet (HFD)-induced hepatic steatosis. (**A**) Body weight gain in response to normal chow diet (NCD) and HFD for FcγRIIb^+/+^ mice, and HFD for FcγRIIb^−/−^ mice (*n* > 5/group). (**B**) Liver weight after 16 weeks of NCD and HFD feeding in FcγRIIb^+/+^ on NCD and HFD, FcγRIIb^−/−^ mice on HFD (*n* > 5/group). (**C**) Liver/body weight ratio of FcγRIIb^+/+^ on NCD and HFD, FcγRIIb^−/−^ mice on HFD for 16 weeks (*n* > 5/group). (**D**) Gross morphology changes in liver of FcγRIIb^+/+^ mice on NCD and HFD, FcγRIIb^−/−^ mice on HFD for 16 weeks. Arrows indicate lipid droplets in liver. (**E**) Liver triglyceride content in FcγRIIb^+/+^ on NCD and HFD, FcγRIIb^−/−^ on HFD for 16 weeks (*n* > 3/group). (**F**) Representative H&E stained (top, 20×) and Oil Red O–stained (bottom,20x) images of liver sections from FcγRIIb^+/+^ mice on NCD and HFD, FcγRIIb^−/−^ mice on HFD for 16 weeks. (**G**) ALT and AST levels in the serum of FcγRIIb^+/+^ and FcγRIIb^−/−^ mice after 16 weeks of HFD (*n* > 5/group). (**H**) qRT-PCR analysis of mRNA expression of *SREBP-1*, *ACC1*, *FASn*, and *CD36*. Total RNA was isolated from FcγRIIb^+/+^ mice fed with NCD, and FcγRIIb^+/+^ or FcγRIIb^−/−^ mice on HFD. The data shown are the means ± SEM. ^#^ *p* < 0.05, control mice (FcγRIIb^+/+^) of NCD vs. HFD; * *p* < 0.05, HFD feeding of FcγRIIb^+/+^ vs. FcγRIIb^−/−^ mice.

To examine the regulatory function of FcγRIIb in hepatic lipid metabolism in vivo, the following experiments were performed in FcγRIIb knockout mice (FcγRIIb^−/−^) and wild type mice (FcγRIIb^+/+^). Wildtype mice were fed with the Normal Chow Diet (NCD) and High Fat Diet (HFD) to confirm if HFD induced hepatic steatosis in an animal model. HFD feeding induced significantly body weight gain (Figure 4A), liver weight gain (Figure 4B), and increased liver/body weight ratio (Figure 4C), compared with NCD. Gross morphology, pathology changes, and triglyceride content increase were shown in Figure 4D,F. This confirmed that HFD feeding induce fatty liver in mice. Under HFD feeding, FcγRIIb^−/−^ mice exhibited no significant body weight gain (Figure 4A), but lower liver weight (Figure 4B) and liver/body weight ratio (Figure 4C, FcγRIIb^+/+^ 0.04489 ± 0.0024 vs. FcγRIIb^−/−^ 0.0341 ± 0.0026). Gross morphology of FcγRIIb^−/−^ mice showed less pale liver (Figure 4D) which indicates less fat accumulation in liver, fewer lipid droplets, and vacuoles (Figure 4F), and lower triglyceride level (Figure 4E, FcγRIIb^+/+^ 3.465 ± 0.171 vs. FcγRIIb^−/−^ 1.555 ± 0.529). Moreover, serum alanine aminotransferase (ALT) and aspartate aminotransferase (AST), two biomarkers for liver injury, were measured. As shown in Figure 4G, serum ALT in FcγRIIb^−/−^ mice were significantly lower than that in FcγRIIb^+/+^ mice, indicating less hepatocyte injury in FcγRIIb^−/−^ mice. Gene mRNA expression was confirmed in the animal study (Figure 4H). The upregulation of the genes of interest (*SREBP-1*, *ACC1*, *FASn*, and *CD36*) in HepG2 cells was verified in FcγRIIb^+/+^ mice fed on NCD, and FcγRIIb^+/+^ or FcγRIIb^−/−^ mice on HFD. HFD feeding resulted an upregulation of the genes of interest, which is supposed to promote lipogenesis and lipid transport. Knockout of FcγRIIb significantly reversed this effect of HFD. These data support that our in vivo finding on the mechanism underlying the effect of FcγRIIb on lipid accumulation reflects the physical event in vivo.

### 2.5. FcγRIIb Decreased Hepatic Insulin Sensitivity In Vitro and In Vivo

**Figure 5:**
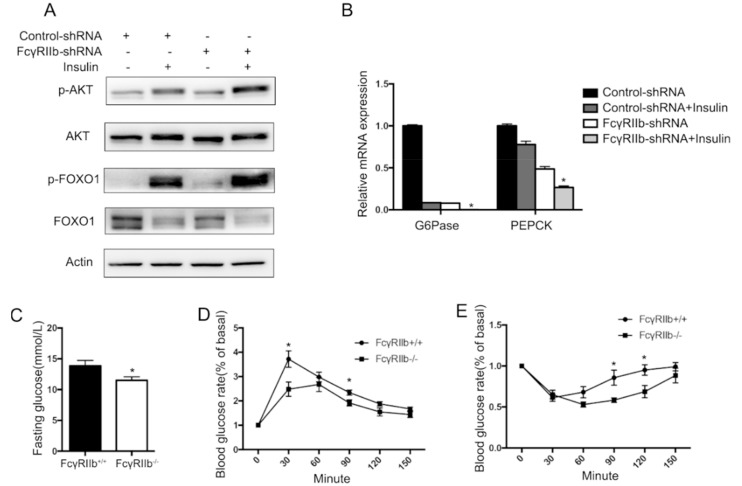

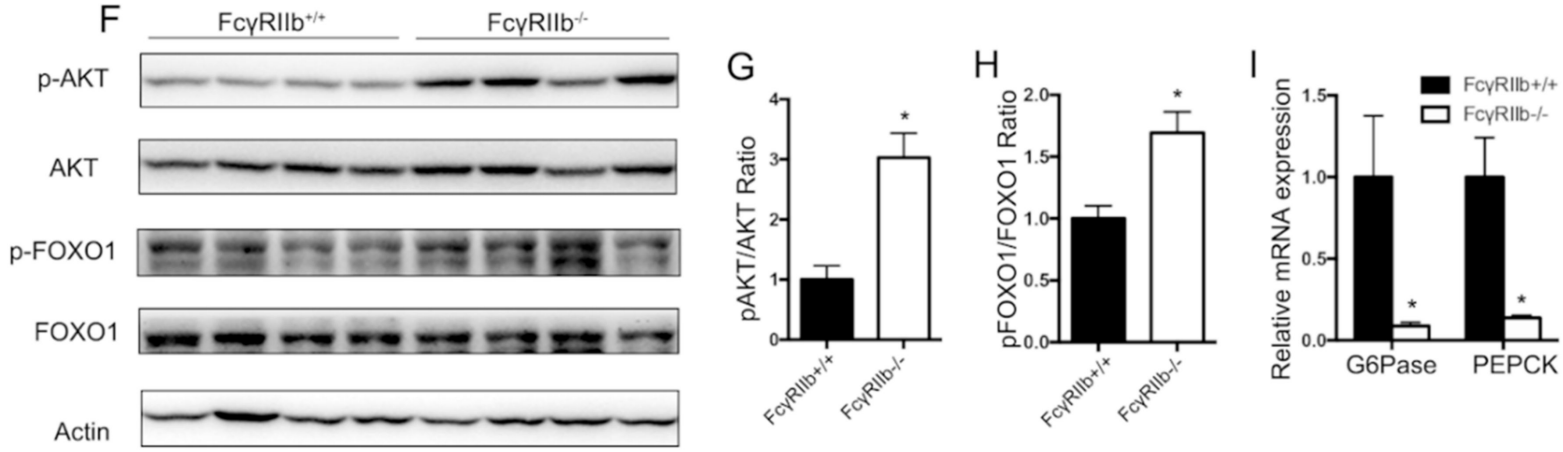
Effect of FcγRIIb on AKT phosphorylation. (**A**) HepG2 cells were starved for 18 h before insulin for 30 min insulin treatment. Protein and RNA were collected for western blot and qRT-PCR. Western blot analysis of insulin-induced AKT and FOXO1 phosphorylation after FcγRIIb shRNA administration. HepG2 were incubated with 100 nmol/L insulin for 30 min after FcγRIIb shRNA or control shRNA administration for 48 h. (**B**) qRT-PCR of mRNA expression of the known downstream genes of FOXO1, *G6Pase*, and *PEPCK*. HepG2 cells were treated the same way as described in A. * *p* < 0.05, between control shRNA and FcγRIIb shRNA pretreatment under insulin activation. (**C**) Blood glucose level under fasting condition in HFD fed FcγRIIb^+/+^ and FcγRIIb^−/−^ mice. (**D**,**E**) GTT and ITT assay of HFD fed FcγRIIb^+/+^ and FcγRIIb^−/−^ mice. (**F**) Western blot analysis of AKT and FOXO1 phosphorylation in vivo. FcγRIIb^+/+^ and FcγRIIb^−/−^ mice were sacrificed and liver were harvested after 16 weeks of HFD. (*n* = 4/group). (**G**,**H**) Statistical analysis of pAKT/AKT ratio and pFOXO1/FOXO1 ratio according to (**F**). (**I**) Relative mRNA expression of *G6Pase* or *PEPCK* was analyzed by qRT-PCR from liver of FcγRIIb^+/+^ and FcγRIIb^−/−^ mice after 16 weeks HFD administration (*n* = 4/group). The data shown are the means ± SEM. * *p* < 0.05.

To investigate the role of FcγRIIb in regulating hepatocyte glucose metabolism, HepG2 cells were treated with or without insulin [15], and the phosphorylation of the known mediators for insulin signaling (AKT and FOXO1) and mRNA levels of *G6Pase* and *PEPCK* (the known downstream factors of FOXO1) were examined. Insulin markedly upregulated phosphorylation of AKT and FOXO1 (lane 2 in Figure 5A), and downregulated *G6Pase* and *PEPCK* mRNA expression (bar 2 in Figure 5B). Whereas knockdown of FcγRIIb with lentivirus-carried FcγRIIb shRNA significantly enhanced the effect of insulin on phosphorylation of AKT and FOXO1 and the consequent mRNA expression of *G6Pase* and *PEPCK* (lane 4 in Figure 5A and bar 4 in Figure 5B), suggesting an inhibiting effect of FcγRIIb on insulin-induced phosphorylation of AKT and FOXO1, and resultant decrease in *G6Pase* and *PEPCK* mRNA. The result that the knockdown of FcγRIIb significantly lowered the basal level of *G6Pase* and *PEPCK* mRNA (bar 3 in Figure 5B) without markedly altering phosphorylation of AKT and FOXO1 (lane 3 in Figure 5A) suggests that FcγRIIb inhibits insulin-suppressed mRNA expression of *G6Pase* and *PEPCK* through more than one mechanism.

To examine if the above finding occurs in vivo, wild type and FcγRIIb^−/−^ mice were tested in the following experiments. Compare with wild type mice, FcγRIIb^−/−^ showed lower serum glucose level after 6 h fasting (Figure 5C), and after glucose or insulin stimulations (Glucose Tolerance Test and Insulin Tolerance Test assay) (Figure 5D,E), suggesting a better insulin sensitivity. Moreover, livers of FcγRIIb^+/+^ and FcγRIIb^−/−^ mice were harvested to detect phosphorylation of AKT and FOXO1, and mRNA expression of *G6Pase* and *PEPCK*. In consistent with our in vitro results, FcγRIIb^−/−^ increased phosphorylation of AKT and FOXO1 (Figure 5F,H), and decreased *G6Pase*, *PEPCK* mRNA expression (Figure 5I), indicating FcγRIIb enhances insulin resistant in HFD.

## 3. Discussion

FcγRIIb, as an IgG receptor, is commonly recognized as being widely expressed in hematopoietic cells. However, recent research demonstrates that FcγRIIb is also expressed in other cells. In this report we show that both primary cultured hepatocytes and HepG2 express *FcγRIIb*. Under OA treatment, HepG2 *FcγRIIb* is increasingly upregulated with increasing dose and time of the treatment. Knockdown of *FcγRIIb* in HepG2 by shRNA suppresses lipid accumulation in the OA induced fatty liver cell model, and downregulates mRNA expression of genes involved in lipogenesis and lipid transport. Consistent with the in vitro results, knockout of FcγRIIb inhibits the development of fatty liver in mice fed with HFD. In glucose metabolism, FcγRIIb suppresses insulin-induced phosphorylation of AKT and FOXO1, while knockdown of FcγRIIb ameliorates insulin-induced AKT and FOXO1 activation, and decreases *G6Pase* and *PEPCK* mRNA expression; these results are recapitulated in vivo.

Hepatocyte function includes lipid and glucose metabolism. Abnormality of lipid and glucose metabolism in hepatocytes promotes the development of fatty liver. Hepatocyte triglyceride synthesis is mainly finished by de novo lipogenesis and fatty acid uptake [3]. In this study we find that FcγRIIb knockout suppresses lipid accumulation in both hepatocytes and the liver, meanwhile suppresses mRNA expression of the *ACC1*, *FASn*, *CD36*, *MTTP* and *ApoB* (known factors for lipid synthesis and transportation) (Figure 3), suggesting that FcγRIIb enhances fatty acid uptake and esterification. Some known signaling pathways including AMP-activated protein kinase (AMPK) [16], Extracellular Regulated protein Kinases(ERK) [17], and p38 [18] are involved in the development of fatty liver. How FcγRIIb affects the expression of genes needs further investigation.

In our study, knockdown of FcγRIIb in HepG2 exhibited a 40% decrease in lipid accumulation (Figure 3D, lane 2 vs. lane 4). Similarly, the protective effect of FcγRIIb^−/−^ showed nearly 70% decrease in lipid accumulation in the liver of HFD induced animal model. This 30% difference between the in vitro and in vivo experiments may be caused by an incomplete knockdown of FcγRIIb in HepG2 cells or a protective effect of FcγRIIb-knockout in other cell types. Considering that the liver tissue is composed of hepatocytes, hepatic stellate cells, Kupffer cells, and sinusoidal endothelial cells, which are all reported to contribute to the development of fatty liver [19], mice with FcγRIIb^−/−^ specific to hepatocytes are the best choice to verify if the mechanism identified in HepG2 exists and acts the same way in vivo. FcγRIIb is an inhibitory receptor specific for IgG, known for regulating immune cells [10]. It is conceivable that FcγRIIb^−/−^ mice may have an inflammatory state, which may alter hepatic lipid and glucose metabolism through immune cells. It has been reported that FcγIIb is expressed in macrophages [20] and B cells [21]. Kupffer cell plays an important role in fatty liver pathology though the role of B cells is unclear [19]. Kupffer cell polarization towards M1 is one of the factors that aggravate simple fatty liver into steatohepatitis [19]. Although FcγIIb is an inhibitory receptor, many studies showed that knockdown of FcγIIb ameliorates inflammation. FcγIIb deficiency improves atherosclerosis by enhancing macrophage M2 polarization and decreasing inflammation [20]. In our study, global FcγRIIb^−/−^ mice showed less hepatic steatosis, which also suggests less inflammation. Nevertheless, bone marrow transplant from FcγRIIb^+/+^ mice to FcγRIIb^−/−^ mice will provide more insight into the effect of white cells on NAFLD. Moreover, a rescue experiment in FcγRIIb^−/−^ mice treated with an FcγRIIb-carrying virus will confirm the effect of FcγRIIb.

It is known that insulin stimulation activates AKT phosphorylation followed by FOXO1 phosphorylation in hepatocytes [4]. FOXO1 is a transcription factor. Phosphorylation of FOXO1 suppresses its translocation from plasma into nuclear, therefore suppresses downstream *G6Pase* and *PEPCK* expression. G6Pase and PEPCK are essential enzymes for gluconeogenesis, which cause blood glucose concentration changes in vivo [5]. Global FcγRIIb deficiency improves insulin sensitivity [13]. Our study here shows that *FcγRIIb* knockdown and knockout increase the insulin-induced phosphorylation of AKT and FOXO1 in HepG2 and the animal model (Figure 5), suggesting that FcγRIIb inhibits insulin-induced activation of AKT and FOXO1, and in turn upregulates *G6Pase* and *PEPCK* in hyperlipemia in hepatocytes. This effect of FcγRIIb decreases insulin sensitivity by increasing glucose generation in fatty liver. The phenomenon that FcγRIIb knockdown causes more inhibition on mRNA expression of *G6Pase* and *PEPCK* without altering the basal level of AKT or FOXO1 phosphorylation (Figure 5B) suggests that AKT is not the only pathway mediating the inhibiting effect of FcγRIIb on mRNA expression of *G6Pase* and *PEPCK*. Our results shown in Figure 5A, *FcγRIIb* shRNA treatment significantly decreased FOXO1 protein (lane 1 and lane 3), indicates that FcγRIIb may regulate FOXO1 expression at mRNA or protein level, or both. By far, no reports in the literature suggest that FcγRIIb regulates mRNA transcription and stability. As for protein translation and stability, Huang et al. reported that post-translational modifications, including phosphorylation and acetylation of FOXO1, cause ubiquitination of FOXO1 in HepG2 [22]. Our current study showed that knockdown of *FcγRIIb* caused a very mild increase in Phospho-FOXO1 and a decrease in total FOXO1, suggesting a mechanism that FcγRIIb protects FOXO1 from ubiquitination. It is conceivable that *FcγRIIb*-knockdown in HepG2 cells does not cause a marked increase in phospho-FOXO1 if phospho-FOXO1 is very unstable. As our current study is not designed to elucidate the molecular details of the effect of FcγRIIb on FOXO1, we did not examine if and how FcγRIIb regulates FOXO1 post-translational modification. Though FcγRIIb as a tyrosine kinase receptor is able to phosphorylate its downstream factors [10], we have no data to support that FOXO1 is a direct downstream factor of FcγRIIb. To our knowledge, it lacks information regarding direct interaction between the proteins. A successful study focusing on the interaction of these tow proteins is needed to clarify the detail of mechanism.

In summary, our study demonstrated the FcγRIIb expression in hepatocyte, and the protective effect of FcγRIIb deficiency on lipid accumulation and insulin resistance in hepatocytes and the liver. Blocking FcγRIIb may be a useful strategy for managing NAFLD.

## 4. Materials and Methods

### 4.1. Primary Hepatocyte Isolation and Culture

Primary mouse hepatocytes were isolated from the livers of male FcγRIIb^+/+^ and FcγRIIb^−/−^ mice (8 weeks old in C57BL/6J background), and the protocol was as previously described [23]. Briefly, mice were anesthetized, and their livers were perfused with 0.5 mg/mL type II collagenase (Sigma-Aldrich, St. Louis, MO, USA), via the inferior vena cava to isolate hepatocytes. Mouse hepatocytes were cultured in RPMI-1640 containing 10% FBS, 100 units/mL penicillin, and 0.1 mg/mL streptomycin. Mouse hepatocytes were ready for experiments after 1–2 days in culture.

### 4.2. Cell Culture

HepG2 cells were purchased from ATCC (Manassas, VA, USA). Modified Eagle’s medium (MEM) was bought from CORNING (10-009CVR). Insulin were from Sigma-Aldrich (I5500-10 mg). C reactive protein (CRP) was bought from APPLYGEN (Beijing, China, C2308). Oleic acid (OA) was purchased from Sigma-Aldrich, O1383.

### 4.3. Animal

Male FcγRIIb^+/+^ and FcγRIIb^−/−^ mice (C57BL/6J background, the Jackson Laboratory, 002848, Sacramento, CA, USA) were housed and maintained on a 12-h-light-dark cycle with a regular unrestricted diet. HF diet (Research Diets, D12108C) were fed since 6 to 8 weeks age, ad libitum, with free access to water. All animal experiments were conducted under protocols approved by the Animal Research Committee of the Institute of Laboratory Animals, Chinese Academy of Medical Sciences, and Peking Union Medical College (ACUC-A01-2014-023, 3 March 2014). Mice were weighted every week. GTT and ITT assay were performed followed standard protocol. After 16 weeks of HFD, mice were sacrificed under anesthetization, mice blood, and tissues were harvested.

### 4.4. RNA Interference

Short-hairpin RNA (shRNA)-encoding DNA sequences were synthesized by TIANYI HUIYUAN (Beijing, China) and constructed into pSIH-H1 plasmids (pSIH-H1, SI501A-1, System Biosciences, Palo Alto, CA, USA). Lentivirus were prepared by using packing plasmid psPAX2 (Addgene#12260) and pMD2.G (Addgene#12259). The sequence of shRNA target sequence was 5′-tgatgaccagaaccgtattta-3′.

### 4.5. Quantitative Real-Time PCR

Total RNA was isolated from cells or pulverized liver using TRIzol (Invitrogen, Carlsbad, CA, USA). RNA reverse transcription was using TIANGEN kit (KR103, Tiangen Biotechnology, Beijing, China). Quantitative real-time reverse-transcriptase PCR (qRT-PCR) was performed using the SYBR Green I Q-PCR kit (TransGen Biotech, Beijing, China) on a Bio-Rad IQ5 system (Bio-Rad, Hercules, CA, USA). All gene expression data were normalized to β-actin expression levels.

### 4.6. Western Blot Analysis

Proteins were extracted from frozen organ samples or cultured hepatocytes in cell lysis buffer, and 30 μg of protein were loaded onto a 10% SDS–polyacrylamide gel and separated proteins were transferred to PVDF membranes. Western blot assays were performed using antibodies specific for p-AKT, AKT, p-FOXO1, FOXO1, and Actin (Cell Signaling Technology, Danvers, MA, USA).

### 4.7. Histology and Immunohistochemistry

For H&E and Oil Red O staining, liver tissue was embedded into OCT and frozen in liquid nitrogen and cut into 6 μm sections. H&E staining and Oil Red O staining were performed followed standard protocol.

### 4.8. Triglyceride Content Assay

Intracellular and liver triglycerides were assayed using a triglyceride assay kit (GPO-POD; Applygen Technologies Inc., Beijing, China). Intracellular and liver triglycerides were assayed using a triglyceride assay kit (GPO-POD; Applygen Technologies Inc., Beijing, China). Intracellular and liver triglycerides were assayed using a triglyceride assay kit (GPO-POD; Applygen Technologies Inc.). Intracellular and liver triglycerides were assayed using a triglyceride kit from APPLYGEN (Beijing, China, E1013-1015).

### 4.9. Statistical Analysis

Data are presented as means ± SEM and were compared between or among groups by ANOVA. *p* < 0.05 was considered statistically significant.

## Abbreviations

NAFLD	Non-alcoholic fatty liver disease
OA	Oleic Acid
CRP	C Reactive Protein
NCD	Normal Chow Diet
HFD	High Fat Diet
